# Identification of Cryptic Promoter Activity in cDNA Sequences Corresponding to PRRSV 5′ Untranslated Region and Transcription Regulatory Sequences

**DOI:** 10.3390/v14020400

**Published:** 2022-02-15

**Authors:** Jayeshbhai Chaudhari, The Nhu Nguyen, Hiep L. X. Vu

**Affiliations:** 1 Nebraska Center for Virology, University of Nebraska-Lincoln, Lincoln, NE 68583, USA; jayeshvet03@gmail.com (J.C.); nhuthe06081995@gmail.com (T.N.N.); 2 School of Veterinary Medicine and Biomedical Sciences, University of Nebraska-Lincoln, Lincoln, NE 68583, USA; 3 Department of Animals Sciences, University of Nebraska-Lincoln, Lincoln, NE 68583, USA

**Keywords:** porcine reproductive and respiratory syndrome virus (PRRSV), 5′ untranslated region (5′ UTR), transcription regulatory sequence (TRS), eukaryotic promoter, nanoluciferase (*nluc*)

## Abstract

To investigate the role of PRRSV nonstructural proteins (nsps) in viral RNA replication and transcription, we generated a cDNA clone of PRRSV strain NCV1 carrying the nanoluciferase (*nluc*) gene under the control of the transcription regulatory sequence 6 (TRS6) designated as pNCV1-Nluc. Cells transfected with the pNCV1-Nluc DNA plasmid produced an infectious virus and high levels of luciferase activity. Interestingly, cells transfected with mutant pNCV1-Nluc constructs carrying deletions in nsp7 or nsp9 regions also exhibited luciferase activity, although no infectious virus was produced. Further investigation revealed that the cDNA sequences corresponding to the PRRSV 5′ untranslated region (UTR) and TRS, when cloned upstream of the reporter gene *nluc*, were able to drive the expression of the reporter genes in the transfected cells. Luciferase signals from cells transfected with a reporter plasmid carrying PRRSV 5′ UTR or TRS sequences upstream of *nluc* were in the range of 6- to 10-fold higher compared to cells transfected with an empty plasmid carrying *nluc* only. The results suggest that PRRSV 5′ UTR and TRS-B in their cDNA forms possess cryptic eukaryotic promoter activity.

## 1. Introduction

Porcine reproductive and respiratory syndrome virus (PRRSV) is the causative agent of a disease of swine characterized by reproductive failure in pregnant sows and respiratory diseases in young pigs. PRRSV is classified into the family *Arteriviridae*, which includes equine arterivirus (EAV), lactate dehydrogenase elevating virus (LDV), and simian hemorrhagic fever virus (SHFV) [1,2]. The PRRSV genome is a positive-sense, single-stranded RNA molecule of approximately 15 kb, which is capped and polyadenylated at its 5′ and 3′ termini, respectively [3]. The 5′ proximal 75% of the viral genome encodes two large overlapping open reading frames (ORFs), ORF1a and ORF1b, where the latter is expressed via a −1 programmed ribosomal frameshift (PRF). ORF1a and ORF1b are translated to produce two large polyproteins, pp1a and pp1b, respectively, which are cleaved by virally encoded proteases to generate 13–16 nonstructural proteins (nsps) [4]. Additionally, there is another PRF located in nsp2, within ORF1a, which signals the −1 and −2 PRF to produce two additional proteins, nsp-2N and nsp2-TF, respectively [5]. Collectively, the nsps are responsible for the replication and transcription of the viral RNA genome, as well as the modulation of the host immune responses (reviewed in [6]). Of these nsps, nsp7 is the most immunogenic protein. Antibodies against nsp7 are consistently detected in infected pigs at around 14 days post-infection, and nsp7 is a target for the serodiagnosis of PRRSV [7]. The nsp9 protein contains the putative RNA-dependent RNA polymerase (RdRp) domain in the C-terminal, and the newly identified nidovirus RdRp-associated nucleotidyltransferase domain in the N-terminal [4,8]. Additional to our knowledge of its polymerase activity, nsp9 has been identified to interact with other viral proteins (e.g., nucleocapsid (N) protein) and cellular proteins, (e.g., annexin A2, retinoblastoma protein, and DEAD box RNA helicase) to regulate the viral replication [9,10,11]. A single mutation within the catalytic domain of nsp9 abolishes its polymerase activity [12].

The remaining 3′ proximal 25% of the viral genome contains eight overlapping ORFs, which encode eight structural proteins. One unique characteristic of PRRSV (and other members of the order *Nidovirales*) is that the structural proteins are expressed from a nested set of sub-genomic mRNAs (sgmRNAs), each of which carries a common 5′ and 3′ untranslated region (UTR) identical to the viral genomic 5′ and 3′ UTR [13]. The sgmRNAs are synthesized via a discontinuous process, in which transcriptional regulatory sequences (TRSs) located both at the viral 5′ UTR (called leader TRS or TRS-L) and at the 5′ end of each structural ORF (called body TRS or TRS-B), are the key elements [13,14]. PRRSV TRS-L contains a hexanucleotide motif, UUAACC, that is conserved among the different PRRSV genotypes [15,16,17]. There are six canonical TRS-B sequences responsible for driving the transcription of the six sgmRNAs, designated as sgmRNAs 2–7. For the transcription of the viral sgmRNAs, the viral transcriptional machinery uses the viral genomic RNA ((+) gRNA) as a template, and initiates the synthesis of the antisense sub-genomic RNA ((−) sgRNA) intermediates from the 3′ terminus. Upon encountering one of the TRS-B sequences, the viral transcriptional machinery, together with the nascent (−) RNA, dissociates and rejoins to the common TRS-L, located in the 5′ UTR, by complementary base-paring between the antisense TRS-B and the sense TRS-L sequence. RNA synthesis is resumed to generate a (−) sgRNA molecule that entirely lacks the ORFs 1a and 1b, as well as one or more structural ORFs, depending on which TRS-B is utilized (reviewed in [18]). The process of disjoining and rejoining is random, leading to the generation of a set of nested (−) sgRNA intermediates of different sizes. The (−) sgRNA7 molecule is the smallest, containing only the ORF7, whereas each of the other (−) sgRNAs contain one additional ORF relative to the next-smaller (−) sgRNA. The (−) sgRNAs in turn serve as templates to produce a set of nested sgmRNAs. 

Reverse genetics has been employed to generate a recombinant PRRSV expressing reporter genes, such as green fluorescence protein (GFP) and luciferase [19,20]. Typically, the reporter gene is inserted into the PRRSV genome in the form of an additional ORF (reviewed in [21]). A common position for the insertion of a reporter gene into the PRRSV genome is the intergenic junction between ORF1b and ORF2a. In this case, the transcription of the reporter gene is driven by a TRS-B sequence located within the ORF1b. Consequently, a synthetic TRS-B sequence must be incorporated into the 3′ end of the reporter gene to regulate the transcription of ORF2a. Another common position for the insertion of a reporter gene is between ORF7 and 3′ UTR. In this case, a synthetic TRS-B sequence needs to be incorporated upstream of the reporter gene to drive its transcription. All six canonical TRS-B sequences have been used to drive the expression of the reporter gene. However, TRS6, which drives the expression of sgmRNA6 responsible for encoding the viral M protein, has been widely used based on initial studies suggesting that this is the stronger TRS [19,22,23].

Our laboratory is interested in studying the role of different PRRSV nsps in the replication and transcription of its RNA genome. We used a DNA-launch reverse genetics approach to generate a cDNA clone based on a type-2 PRRSV isolate designated as pNCV1. To facilitate the detection of successful sgmRNA transcription, the nanoluciferase (*nluc*) gene was inserted into the pNCV1 cDNA in the form of an additional ORF, under the control of a synthetic TRS6 sequence. Cells transfected with the pNCV1-Nluc DNA plasmid expressed high levels of luciferase activity. Interestingly, cells transfected with mutant pNCV1-Nluc DNA plasmids, in which the nsp7 or nsp9 was deleted, also exhibited a luciferase signal, suggesting that the expression of the *nluc* gene was independent of the virally encoded RNA replicase. Considering that the *nluc* gene was inserted into the NCV1 genome immediately downstream of the synthetic TRS6 sequence, and the cells were transfected with a DNA plasmid, we hypothesized that the cDNA sequence of TRS6 might be able to drive the expression of the *nluc* gene. We describe our results, demonstrating that the cDNA forms of arterivirus 5′ UTR and PRRSV TRS sequences contain a previously unidentified eukaryotic promoter.

## 2. Materials and Methods

### 2.1. Cells, Antibodies, and Reagents

MARC-145 cells were maintained in Dulbecco’s Modified Eagle Medium (DMEM) containing low glucose and low bicarbonate, while human embryonic kidney (HEK) 293T cells (ATCC^®^ CRL-3216^TM^) were maintained in DMEM high-glucose (Life Technologies, Grand Island, NY, USA). All media were supplemented with 10% fetal bovine serum (FBS; Sigma, St. Louis, MO, USA), 100 units/mL of penicillin, and 100 μg/mL of streptomycin (Sigma, St. Louis, MO, USA) (herein designated as cDMEM). All cell lines were cultured at 37 °C in 5% CO_2_ in an incubator. Mouse monoclonal antibody SDOW17, specific to PRRSV-N protein, was purchased from the National Veterinary Services Laboratories (Ames, IA, USA). Alexa Fluor-488 F(ab’)_2_ fragment of goat anti-mouse IgG (H+L) antibody was purchased from Invitrogen (Eugene, OR, USA). Goat anti-mouse IgG (H+L)-HRP antibody and DAPI (4′,6-diamidino-2-phenylindole dihydrochloride) were purchased from Thermo Fisher Scientific (Carlsbad, CA, USA). 

### 2.2. Viruses and Full-Length cDNA Plasmids 

PRRSV strain NCV1 was isolated from the serum samples collected from three-week-old piglets from a farm that broke with PRRSV. The virus was consecutively passaged in MARC-145 cells for 95 passages, followed by three rounds of plaque purification. Two separate viral plaques were picked, and the virus was amplified in MARC-145 cells. For this study, the virus used was derived from the plaque clone-1.

The infectious clone for PRRSV-1 strain SD01-08 (pSD0108) was kindly provided by Dr. Fang (University of Illinois at Urbana-Champaign) [24]. The infectious clone for EAV was provided by Dr. Balasuriya (Louisiana State University) [25].

### 2.3. Construction of NCV1 Infectious cDNA Clone pNCV1

**Figure 1:**
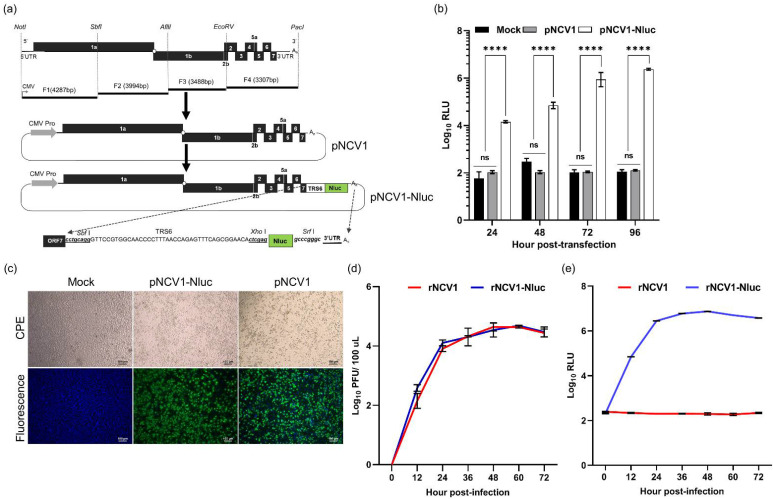
Construction and in vitro characterization of the cDNA infectious clone of PRRSV expressing luciferase reporter gene. (**a**) The overall cloning strategy to construct pNCV1 and pNCV1-Nluc full-genome cDNA clone. Top: A schematic representation of NCV1 genome and restriction enzymes used for assembling the full-length clone. Horizontal lines indicate four PCR fragments (F1–F4), together with their size. Middle: A schematic representation of the full-length pNCV1 plasmid in which the four PCR fragments were sequentially assembled into the pUC19 plasmid, downstream of the CMV promoter. Bottom: A schematic representation of the pNCV1-Nluc plasmid in which the *nluc* reporter gene was inserted into the pNCV1 plasmid between ORF7 and 3′ UTR. Two enzyme sites, *Sbf*I and *Xho*I, and the TRS6 were inserted before the *nluc* gene, and another *Srf*I enzyme site was inserted after *nluc*. (**b**) Luciferase signals from MARC-145 cells transfected with the indicated plasmids. Data are expressed as log_10_ relative light units (RLU). (**c**) Representative images of MARC-145 cells transfected with the plasmids and subjected to an IFA for detection of N protein expression at 96 hpt. ns: non-significant, **** *p* ≤ 0.0001. (**d**) Multiple-step growth curves of the rNCV1 and rNCV1-Nluc in MARC-145 cells. (**e**) Luciferase signals from MARC-145 cells infected with the indicated recombinant viruses at an MOI of 0.1.

Viral RNA was isolated from the plaque clone-1 virus using the QIAmp viral RNA isolation kit (Qiagen GmbH, Qiagen Strasse 1, Hilden, Germany). cDNA was synthesized using Superscript^TM^ IV reverse transcriptase kit (Invitrogen, Vilnius, Lithuania). Overlapping PCR fragments encompassing the full-length sequence of the NCV1 genome were amplified using four pairs of primers listed in Table 1. Each PCR fragment was flanked by a unique pair of restriction enzyme sites naturally occurring within the NCV1 genome (Figure 1). Additionally, two enzyme sites, *Not*I and *Pac*I, were incorporated into the 5′ and 3′ termini of fragments 1 and 4, respectively, to facilitate the cloning of these two PCR fragments. The four PCR amplicons were first cloned into the pJET1.2 blunt-end cloning vector (Thermo Fisher Scientific, Vilnius, Lithuania), and five clones for each fragment were sequenced. The clone that exhibited the consensus sequence of the five clones was selected to assemble a full-length cDNA clone.

To assemble a full-length NCV1 cDNA clone, we first generated a shuttle plasmid (pUCMV) by inserting the cytomegalovirus (CMV) immediate-early promoter, followed by unique restriction enzyme sites *Not*I, *Sbf*I, *Afl*II, *EcoR*V, and *Pac*I, and the hepatitis D virus ribozyme (HDRz) sequence, into the pUC19 plasmid. Next, the four overlapping PCR amplicons were digested from the respective pJET1.2 plasmids and sequentially cloned into the pUCMV plasmid, following standard molecular cloning. The resulting plasmid carrying the full-length cDNA of the PRRSV strain NCV1 was designated as pNCV1.

### 2.4. Construction of pNCV1-Nluc Plasmid

The reporter gene *nluc* was inserted into the pNCV1 cDNA clone at the junction between ORF7 and 3′ UTR. A DNA fragment containing a portion of ORF7 from the enzyme site *Xba*I to the end of ORF7, the synthetic TRS6, *nluc*, the 3′ UTR, and a polyA tail (50 A residues), was synthesized by Synbio Technologies (Monmouth, NJ, USA). This synthetic DNA fragment was cloned into the pNCV1 plasmid between the two enzyme sites *Xba*I and *Pac*I. The resulting plasmid was designated as pNCV1-Nluc.

### 2.5. Construction of NCV1 Carrying Substitution or Deletion in Its Nonstructural Proteins

To generate the pNCV1-Nluc nsp7 mutants, the NCV1 nsp7 was either replaced by the nsp7 of the PRRSV-1 strain SD0108 (designated as pNCV1-Nluc-SD0108-nsp7) or the nsp7 of the EAV strain Bucyrus (designated as pNCV1-Nluc-EAV-nsp7). Primers are listed in Table 2. To construct the pNCV1-Nluc-SD0108-nsp7 plasmid, two PCR fragments were generated. The first PCR fragment (NCV1SD0108nsp7 F1), spanning from the enzyme site *EcoR*I in nsp6 to the end of nsp7 of NCV1, together with 20 nt of pSD0108 nsp7, was amplified from the pNCV1-Nluc plasmid template using the primer pair NCV18601F and SD0108nsp7R. The second PCR fragment (NCV1SD0108 F2) containing 20 nt of the pSD0107 nsp7 and the NCV1 nsp8, followed by the *Pme*I enzyme site, was amplified from the pNCV1-Nluc plasmid template using the primer pair SD0108nsp7F and NCV110875R. Subsequently, NCV1SD0108nsp7 F1 and NCV1SD0108nsp7 F2 were used as megaprimers, and pSD0108 plasmid as a template, to PCR amplify the fragment containing complete SD0108nsp7, which was then cloned into the pNCV1-Nluc using the *EcoR*I and *Pme*I sites. A similar approach was used to generate the pNCV1-Nluc-EAV-nsp7 plasmid.

To delete the nsp7, two overlapping PCR fragments were amplified from the pNCV1-Nluc plasmid, using the primer pairs NCV18601F, Δnsp7R and Δnsp7F, NCV110875R, respectively. These two overlapping fragments were then fused together to generate a fragment spanning from the *EcoR*I to *Pme*I enzyme sites, but lacking the entire nsp7 coding sequence (from aa 2069 to 2328 in the pp1a). The resulting fragment was cloned into the pNCV1-Nluc using the enzyme sites *EcoR*I and *Pme*I to generate the plasmid pNCV1-Nluc-Δnsp7.

To delete the nsp9, two overlapping PCR fragments were amplified from the pNCV1-Nluc plasmid, using the primer pairs NCV19176F, Δnsp9R and Δnsp9F, 12752R, respectively. The overlapping fragments were then fused together to generate a fragment spanning from the *Avr*II site to the *EcoR*V, but lacking 386 aa (from aa 2555 to 2941) of nsp9. The resulting fragment was then cloned into the pNCV1-Nluc plasmid using the enzyme site *Avr*II and *EcoR*V to generate the plasmid pNCV1-Nluc-Δnsp9.

### 2.6. DNA Transfection to Recover Recombinant PRRSV

To recover the recombinant virus from the cDNA clones, MARC-145 cells were seeded in a 6-well plate at the cell density of 5 × 10^5^ cells per well. One day later, the cells were transfected with 2.5 μg of plasmid, using 7.5 μL of Transit-X2 (Mirus Bio LLC, Madison, WI, USA). Transfected cells were monitored for cytopathic effect (CPE). At 96 h post-transfection (hpt), when obvious CPE was observed, cell culture supernatant containing infectious virus was harvested and stored at −80 °C for future use.

### 2.7. Evaluation of Nluc and ORF7 Expression

MARC-145 cells were seeded in a 24-well plate (Greiner Bio-One, Monroe, NC, USA) at the density of 5 × 10^4^ cells per well. One day later, the cells were transfected with 500 ng of DNA plasmid mixed with 1.5 μL of PEI transfectant reagent in 50 μL of Opti-MEM reduced serum medium (Life Technologies, Grand Island, NY, USA). Samples of culture supernatant were collected at various time points post-transfection to evaluate luciferase activity using the Nano-Glo^®^ Luciferase Assay System (Promega, Madison, WI, USA). Luminescence was measured with a Synergy^LX^ multi-mode reader (BioTek, VT, USA). To detect viral ORF7 expression, at 96 hpt, cells were washed twice with 1× phosphate-buffered saline (PBS, pH 7.4), and subsequently fixed and permeabilized using cold methanol: acetone (1:1 *v*/*v*) and subjected to an indirect immunofluorescence (IFA) assay using the SDOW17 anti-N-protein antibody (diluted 1:500 in PBS) and Alexa Fluor-488 F(ab’)_2_ fragment of goat anti-mouse IgG (H+L) antibody (diluted 1:1000 in PBS). Nuclear staining was performed using DAPI diluted 1:4000 in PBS for 7 min at RT. Fluorescence images were taken using a Nikon Eclips Ts2R-FL operated by Nikon NIS Elements (ver 5.02). All images are taken separately at 10× using the GFP and DAPI channels. Images for each filter channel were then overlaid to generate the final image.

### 2.8. Multiple-Step Growth Curve

To assess the viral growth kinetics, MARC-145 cells cultured in 24-well plates were inoculated with rNCV1 or rNCV1-Nluc at a multiplicity of infection (MOI) of 0.1 TCID_50_ per cell. After 1 h adsorption, the viral inoculum was removed, the cell monolayer was washed to remove unbound virus particles, and 500 µL fresh cDMEM was added into each well. Culture supernatants were harvested at various time points post-infection (0, 12, 24, 36, 48, 60, and 72 hpi), and virus titers were determined by plaque assay on MARC-145 cells. 

### 2.9. Construction of Nluc Reporter Plasmids

The pUC19 plasmid was modified to remove the lac promoter and LacZa gene using the primer pair pUC19F and pUC19R, which contained *EcoR*I and *Sph*I enzyme sites, respectively (Table 3). The *nluc* gene was PCR amplified from the pNCV1-Nluc template using the primer pair NlucF and NlucR, which also contained *EcoR*I and *Sph*I. The two PCR amplicons were ligated together to generate the reporter plasmid pUC19-Nluc or empty vector. Five PRRSV TRSs (TRS2, 4, 5, 6, and 7) were synthesized as an oligo containing *EcoR*I site and 20 nt overlapping sequence with *nluc* (Table 3). All five TRSs were used as forward primers and pUC19R (also containing *EcoR*I site) as a reverse primer, to amplify circular plasmid from a pUC19-Nluc template. The resulting PCR fragments were then circularized by digestion with *EcoR*I restriction enzyme, followed by a T4 ligation. TRS3 was commercially synthesized and cloned into pUC19 vector using Gibson assembly cloning procedure as per the manufacturer’s recommendation.

5′ UTR fragments of the arterivirus, including the PRRSV (NCV1), EAV (DQ846750.1), and LDV (NC_001639.1), were commercially synthesized and cloned into pUC19-Nluc plasmid following *EcoR*I and *Sph*I restriction digestion. All cloned plasmids were sequence verified for unwanted mutational changes. 

### 2.10. Construction of PRRSV 5′ UTR Deletion Mutants

PRRSV 5′ UTR hexanucleotide (UUAACC) TRS-L, and six serial 30 nt deletion mutants were constructed using the primer pairs listed in Table 4. Briefly, the seven different blunt-end PCR fragments containing serial 30 nt 5′ UTR deletion mutants were PCR amplified using the pUC19-5′ UTR-Nluc as a DNA template. The blunt-end fragments were then circularized using the Fast DNA end repair kit (Thermo Fisher Scientific, Carlsbad, CA, USA) as per the manufacturer’s recommendation.

### 2.11. DNA Transfection to Evaluate Promoter Activity

HEK-293T cells were seeded in 24-well plates at the density of 2 × 10^5^ cells per well. The following day, the cells were transfected with either an empty plasmid pUC19-Nluc without any promoter, or with pUC19-Nluc containing 5′ UTR or TRS sequences. Each well was transfected with 500 ng DNA plasmid using 1.5 μL PEI. Samples of culture medium were collected at various time points post-transfection, and luciferase activity was measured using the Nano-Glo Luciferase assay kit (Promega, Madison, WI, USA) following the manufacturer’s recommendation.

### 2.12. Statistical Analysis

Data were expressed as mean ± SE from three independent experiments. Statistical analysis was performed using GraphPad prism software version 8.3.1 (GraphPad Software, LLC, San Diego, CA, USA). Comparisons of mean ± SE of two or more groups at one time point were analyzed using ordinary one-way analysis of variance (ANOVA) followed by Dunnett’s multiple comparison test. Mean ± SE of two or more groups at multiple time points was analyzed using two-way analysis of variance (ANOVA) follow by Sidak’s multiple comparison test. For all comparisons, a *p* value < 0.05 was considered significant.

## 3. Results

### 3.1. Generation of a cDNA Infectious Clone of PRRSV Expressing Luciferase Reporter Gene

As a part of our effort to develop a new generation of PRRSV vaccine, we generated an infectious cDNA clone of a type-2 PRRSV strain, designated as NCV1, by cloning the full-length viral cDNA genome into a bacterial plasmid, immediately downstream of the human cytomegalovirus (hCMV) promoter (Figure 1a). To facilitate real-time monitoring of viral infection, the reporter gene *nluc* was inserted into the pNCV1 cDNA genome as an additional ORF between ORF7 and 3′ UTR, under the control of TRS6 (Figure 1a). MARC-145 cells transfected with pNCV1-Nluc plasmid exhibited an elevated level of luciferase signal, which could be detected as early as 24 hpt and peaked at 96 hpt (Figure 1b). On the other hand, cells transfected with the pNCV1 plasmid exhibited basal levels of luciferase signal. At 96 hpt, obvious CPE was observed in cells transfected with either pNCV1 or pNCV1-Nluc, clearly demonstrating that these two cDNA clones were fully infectious (Figure 1c). The recombinant virus recovered from cells transfected with pNCV1 or pNCV1-Nluc, designated as rNCV1 and rNCV1-Nluc, respectively, replicated efficiently in MARC-145 cells. The two recombinant PRRSV strains displayed similar growth kinetics, with a peak viral titer of 4.67 log_10_ pfu/100 µL detected at 60 hpi (Figure 1d). Intense luciferase signals were detected from cells infected with rNCV1-Nluc, starting from 12 hpi and peaking at 60 hpi (Figure 1e). On the other hand, only basal levels of luciferase signals were detected from cells infected with the rNCV1 virus. In summary, we successfully constructed an infectious cDNA clone based on the PRRSV strain NCV1, and a derivative cDNA clone that expresses the reporter gene *nluc*.

### 3.2. Alterations of nsp7 and nsp9 of the PRRSV-Nluc Did Not Affect Nluc Gene Expression

**Figure 2:**
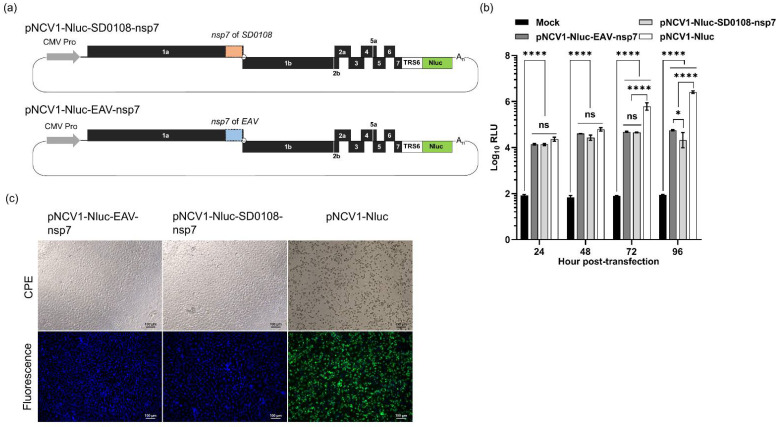
Construction of chimeric PRRSV mutant plasmids. (**a**) Schematic representation of pNCV1-Nluc-nsp7 mutant plasmids in which it nsp7 was either replaced by nsp7 of PRRSV-1 (SD0108) or EAV (Bucyrus). (**b**) MARC-145 cells were transfected with one of the indicated plasmids and luciferase signals were measured at indicated hours post-transfection (hpt). ns: non-significant, * *p* < 0.05, **** *p* ≤ 0.0001. (**c**) Representative images of MARC-145 cells transfected with the indicated plasmids and subjected to an IFA for detection of N protein expression at 96 hpt.

The reporter cDNA infectious clone pNCV1-Nluc was used as a tool to study the roles of different PRRSV nsps in the replication and transcription of the viral genome. We first constructed chimeric pNCV1 cDNA clones by replacing its nsp7 gene with the corresponding genes of a PRRSV-1 strain or an EAV strain. The resulting chimeric cDNA clones were designated as pNCV1-Nluc-SD0108-nsp7 and pNCV1-Nluc-EAV-nsp7, respectively (Figure 2a). MARC-145 cells transfected with the chimeric cDNA clones expressed high levels of luciferase signal, suggesting that sgmRNA synthesis was not affected by the substitution of the viral nsp7 (Figure 2b). However, infectious virus was not recovered either from cells transfected with pNCV1-Nluc-SD0108-nsp7, or those transfected with pNCV1-Nluc-EAV-nsp7 chimeric plasmids (Figure 2c). 

**Figure 3:**
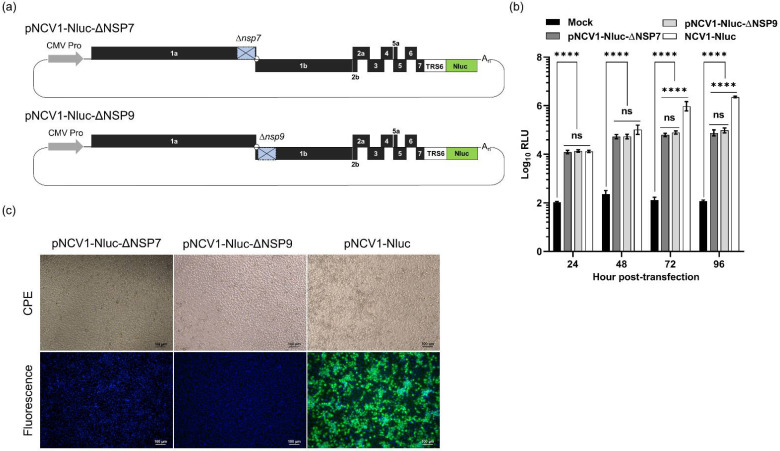
Construction of PRRSV nsp7 and nsp9 deletion mutants. (**a**) Schematic representation of pNCV1-Nluc-ΔNSP7 and ΔNSP9 plasmids in which nsp7 or nsp9 were deleted from the genome. (**b**) MARC-145 cells were transfected with one of the indicated plasmids and luciferase signals were measured at 12, 24, 36, 48, and 96 hpt. ns: non-significant, **** *p* ≤ 0.0001. (**c**) Representative images of MARC-145 cells transfected with the indicated plasmids and subjected to an IFA for detection of N protein expression at 96 hpt.

To further evaluate the role of nsp7 in viral sgmRNA synthesis, we generated a deletion construct by removing the entire nsp7 from the pNCV1-Nluc cDNA clone (Figure 3a). Cells transfected with the pNCV1-Nluc-Δnsp7 still exhibited modest levels of luciferase activity, suggesting that nsp7 is not required for the expression of *nluc* in the pNCV1 plasmid. We then constructed another deletion construct by removing a portion of the viral nsp9, an RNA-dependent RNA polymerase. Surprisingly, cells transfected with the pNCV1-Nluc-Δnsp9 plasmid exhibited slightly lower luciferase signal than those transfected with pNCV1-Nluc, although significantly higher than the mock-transfected cells (Figure 3b). No sign of CPE was observed from cells transfected with either pNCV1-Nluc-Δnsp7 or pNCV1-Nluc-Δnsp9 plasmid, whereas clear CPE was observed from cells transfected with pNCV1-Nluc (Figure 3c). Collectively, the data suggest that the expression of *nluc* from pNCV1-Nluc-Δnsp7 or pNCV1-Nluc-Δnsp9 plasmids was independent of viral RdRp activity.

### 3.3. PRRSV TRS-B cDNA Sequence Possesses Cryptic Promoter Activity

**Figure 4:**
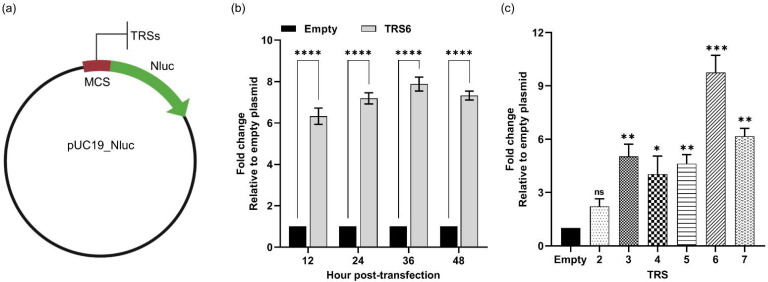
PRRSV TRS-Bs showed cryptic promoter activity. (**a**) Schematic representation of pUC19-Nluc vector with TRS-Bs upstream of Nluc. (**b**) HEK-293T cells were transfected with either an empty reporter plasmid carrying nluc only (Empty) or a plasmid carrying TRS6 upstream of Nluc (TRS6). Luciferase signals were measured at 12, 24, 36, and 48 hpt. (**c**) HEK-293T cells were transfected either with an empty plasmid, or using a reporter plasmid carrying one of the six TRS-B sequences (TRS 2–7). Luciferase signals were measured at 48 hpt. Data are expressed as relative fold change of luciferase signal in cells transfected with plasmids carrying TRS-B compared to cells transfected with an empty plasmid. ns: non-significant, * *p* < 0.05, ** *p* ≤ 0.01, *** *p* ≤ 0.001, **** *p* ≤ 0.0001.

Since *nluc* was cloned immediately downstream of the TRS6 cDNA sequence, we hypothesized that TRS6 might exhibit eukaryotic promoter activity. To test this hypothesis, the PRRSV TRS6 cDNA sequence was cloned into a pUC19 plasmid containing the *nluc* gene, but that was otherwise devoid of any known eukaryotic promoter sequence (Figure 4a). Cells transfected with the pUC19-TRS6-Nluc exhibited a 6–8-fold higher luciferase signal than those transfected with an empty plasmid (Figure 4b). The data suggest that the cDNA sequence corresponding to PRRSV TRS6 is capable of regulating the expression of the *nluc* gene. The PRRSV genome carries six canonical TRS-B sequences, which regulate the transcription of six canonical sgmRNAs (designated as sgmRNA 2–7, respectively). To determine whether other TRS-B cDNA sequences also exhibit promoter activity, the cDNA sequences corresponding to the remaining five TRS-Bs were separately cloned into the reporter plasmid upstream of the *nluc* gene. Cells transfected with the reporter plasmid carrying TRS2 did not exhibit significant levels of luciferase signal compared to those transfected with an empty plasmid (Figure 3c). On the other hand, cells transfected with a reporter plasmid carrying either TRS3, TRS4, TRS5, TRS6, or TRS7 exhibited weak promoter activity, with luciferase signals between 4.04- and 9.73-fold higher compared to cells transfected with an empty plasmid (Figure 4c). TRS6 exhibited the strongest promoter activity at all time points, with the luciferase signal varying between 9- and 10-fold higher compared to cells transfected with an empty plasmid (Figure 4c).

### 3.4. PRRSV 5′ UTR cDNA Sequence Possesses Cryptic Promoter Activity

**Figure 5:**
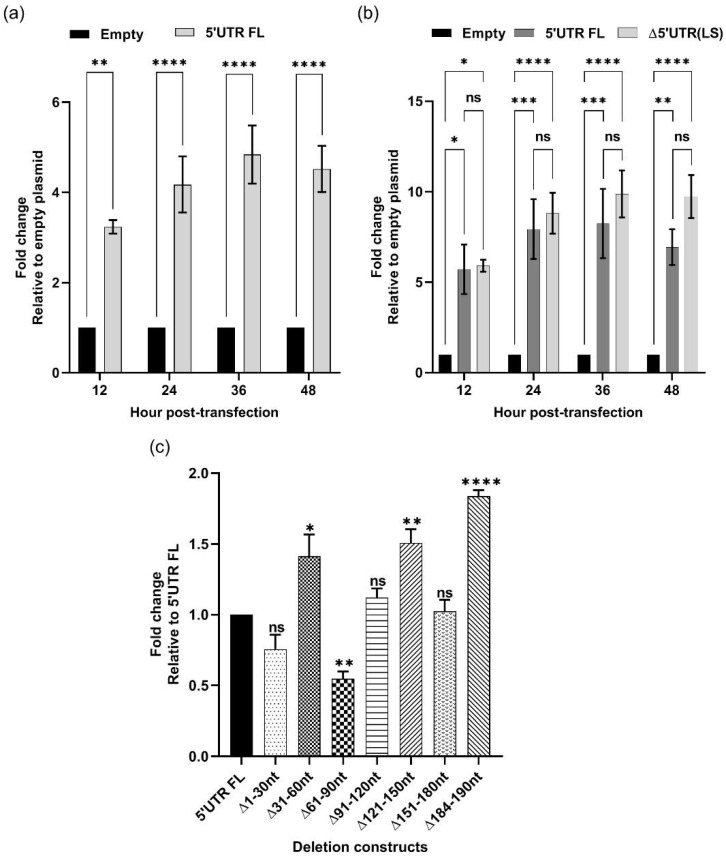
PRRSV 5′ UTR cDNA sequence possesses cryptic eukaryotic promoter activity. (**a**,**b**) HEK-293T cells were transfected with either an empty pUC19-Nluc plasmid (Empty), a plasmid carrying an intact PRRSV 5′ UTR cDNA sequence (5′ UTR FL), or a plasmid carrying 5′ UTR without the hexanucleotides corresponding to the TRS-L (∆5′ UTR LS). Luciferase signals were measured at 12, 24, 36, and 48 hpt. Data are expressed as relative fold changes compared to empty plasmid. (**c**) HEK-293T cells were transfected with serial 30 nt deletion mutants of 5′ UTR, and luciferase signals were measured at 48 hpt. Data expressed as relative fold change in luciferase signal compared to cells transfected with full-length, intact 5′ UTR. ns: non-significant, * *p* < 0.05, ** *p* ≤ 0.01, *** *p* ≤ 0.001, **** *p* ≤ 0.0001.

It has been reported that the hepatitis C virus (HCV) and the dengue virus 5′ UTR cDNA sequence exhibits promoter activity [26,27]. Since we observed that five out of six PRRSV TRS-B cDNA sequences exhibited promoter activity, we wanted to determine whether PRRSV 5′ UTR could also possess promoter activity. The cDNA sequence corresponding to PRRSV 5′ UTR was cloned into the pUC19-Nluc reporter plasmid, upstream of the *nluc* gene. Cells transfected with the pUC19-5′ UTR-Nluc plasmid showed between 3- and 4-fold increases in luciferase signal compared to those transfected with an empty vector (Figure 5a), indicating that the cDNA form of the PRRSV 5′ UTR sequence possesses weak promoter activity.

To locate the sequence responsible for the promoter activity in PRRSV 5′ UTR, we first deleted the highly conserved hexanucleotides corresponding to the TRS-L. Surprisingly, the deletion of the TRS-L from the 5′ UTR did not affect the level of *nluc* expression (Figure 5b), indicating that it was not required for promoter activity. Subsequently, we constructed six deletion mutants by serially removing a stretch of 30 bp from the 5′ UTR. Deletions of 61–90 nt significantly reduced *nluc* expression, with the luciferase signals observed from cells transfected with the Δ61–90 nt construct were only 50% of those exhibited by cells transfected with the intact 5′ UTR plasmid. Interestingly, deletions of 121–150 nt and 184–190 nt resulted in increased expression of Nluc compared to the intact 5′ UTR plasmid (Figure 5c). Collectively, the results suggest that the stretch of 30 nt from position 61 to 90 of the PRRSV 5′ UTR cDNA sequence plays an important role in its promoter activity.

### 3.5. Arterivirus 5′UTR cDNA Sequence Possesses Cryptic Promoter Activity

**Figure 6:**
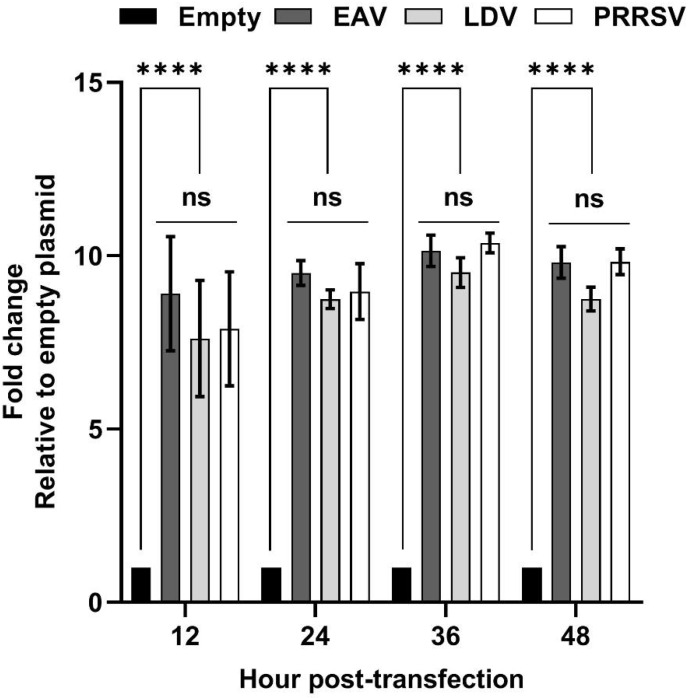
Arterivirus 5′ UTR cDNA sequence exhibits cryptic promoter activity. HEK-293T cells were transfected with either an empty plasmid carrying only Nluc, or plasmids carrying 5′ UTR of PRRSV, EAV, or LDV, cloned in pUC19 vector upstream of Nluc. Luciferase signals were measured at 12, 24, 36, and 48 hpt. Data are expressed as fold change relative to empty vector. ns: non-significant, **** *p* ≤ 0.0001.

We were curious to as to whether the cDNA sequences corresponding to the 5′ UTR of other members of the family *Arteriviridae* also exhibit promoter activity. Therefore, cDNA sequences of EAV and LDV 5′ UTR were cloned into the reporter plasmid, upstream of *nluc*. HEK-293T cells transfected with plasmids carrying the EAV or the LDV 5′ UTR cDNA sequence exhibited between 7- and 9-fold increases in luciferase signal compared to those produced by cells transfected with the empty plasmid (Figure 6). No significant differences were observed regarding the luciferase signals between the cells transfected with plasmids carrying 5′ UTR of PRRSV, EAV, or LDV. 

## 4. Discussion

The reverse genetics system is a powerful tool for engineering the viral RNA genome. The first reverse genetics system for PRRSV was reported in 1998 [28]. Since then, many infectious cDNA clones for PRRSV have been constructed. Two different approaches have been used to construct an infectious cDNA clone for PRRSV (reviewed in [21]). For RNA-based transfection, the full-length viral cDNA genome is cloned downstream of a bacterial phage promoter. In vitro transcription is used to produce the viral RNA transcript, which is then transfected into permissive cells to produce the infectious virus. For DNA-based transfection, the viral cDNA genome is cloned under a polymerase II promoter, and the resulting DNA plasmid containing the viral cDNA genome is transfected into cells without the need of in vitro transcription. The pNCV1 infectious cDNA clone used in this study was generated following DNA-based transfection. Additionally, a reporter gene, *nluc*, was inserted into pNCV1 in the form of an additional ORF, under the control of the TRS6 sequence, so that luciferase activity could be used as an indicator of successful viral sgmRNA synthesis. In this study, high levels of luciferase activity were detected from cells transfected with a full-genome pNCV1-Nluc. Surprisingly, a luciferase signal was also detected from cells transfected with a plasmid carrying a deletion in the nsp7 or nsp9 region (pNCV1-Nluc-Δnsp7 and pNCV1-Nluc-Δnsp9), suggesting that *nluc* expression was independent from the virally encoded RNA replicase. Considering that the cells were transfected with a DNA plasmid, and that the *nluc* gene was cloned immediately downstream of the TRS6 cDNA sequence, we hypothesized that the TRS6 cDNA sequence might possess eukaryotic promoter activity. Using a reporter plasmid, we were able to demonstrate that the TRS6 cDNA sequence could drive the transcription of the *nluc* gene in HEK-293T cells. Further analysis led us to discover that, in addition to PRRSV TRS6, promoter activity is also exhibited by TRS3, TRS4, TRS5, TRS7, and the viral 5′ UTR. 

There are six canonical TRS-B sequences that drive the transcription of six sgmRNAs responsible for encoding the structural genes of PRRSV. All six TRS-B sequences have been evaluated for their ability to drive the expression of the additional ORF inserted into the PRRSV genome [29]. For sgmRNAs 2, 3, and 6, only one corresponding TRS-B site has been identified, whereas for sgmRNAs 4, 5, and 7, two TRS-B sites have been identified [30]. TRS-4.1, TRS-5.1, and TRS-7.1 are used predominantly over TRS-4.2, TRS-5.2, and TRS-7.2, respectively. Only the dominant forms of TRS-4, TRS-5, and TRS-7 have been evaluated for their ability to drive the expression of a reporter gene in the context of an infectious PRRSV cDNA clone [29]. In this study, we chose to evaluate eukaryotic promoter activity of the dominant form of TRS-4 and TRS-5, whereas for TRS7, the less dominant form, TRS-7.2, was selected for promoter activity evaluation. Thus, we do not know whether the other forms of TRS4, TRS5, and TRS7 also exhibit promoter activity.

Recombinant reporter PRRSV strains have been widely used for studying viral protein localization and trafficking, and the detection of viral infection. The synthetic TRS6 sequence is commonly used to regulate the expression of the reporter gene in the PRRSV genome. In the present study, we generated an infectious cDNA clone of PRRSV carrying the reporter gene, *nluc*, in the form of an additional ORF, under the regulation of TRS6. Our goal was to use this construct as a tool to study the roles of different PRRSV nsps in mediating viral sgmRNA synthesis, with the assumption that the expression of *nluc* from the cDNA clone should only be mediated by viral RNA replicase. However, we observed in this study that TRS6 in its cDNA form can drive expression of the reporter gene independent of viral RdRp activity. Therefore, for the purposes of studying the viral, nonstructural proteins involved in mediating the expression of sgmRNA, TRS2 should be chosen to drive the expression of reporter genes to minimize the eukaryotic promoter-mediated expression of reporter genes.

Promoter activity seems to be common in arteriviruses because the 5′ UTR cDNA sequences of EAV and LDV can also drive the expression of the reporter gene, *nluc* (Figure 6). All arteriviruses carry a conserved leader TRS hexanucleotide in their 5′ UTR; for instance, EAV contains the sequence UCAACU [31], LDV contains the sequence UAUAACC [32], and SHFV contains the sequence UUAACC [33]. However, the removal of the hexanucleotide sequence (UUAACC) from the 5′UTR of PRRSV did not significantly affect its ability to drive the expression of *nluc*. Furthermore, all six TRS-Bs of PRRSV used in this study also carry the conserved hexanucleotide sequence. Despite this, TRS2 did not exhibit noticeable levels of promoter activity. Together, the results indicate that the promoter activity of the PRRSV 5′ UTR and the TRS-B are independent of the hexanucleotide sequence (UUAACC). The sequential deletion of nucleotide stretches within the 5′ UTR revealed that the nucleotide residues from 61 to 90 nt are critical for promoter activity. 

Promoter activity has been observed in the cDNA form of the hepatitis C virus (HCV) 5′ UTR and the 5′ end of the flavivirus genome [26,34]. For HCV, the promoter activity of its 5′ UTR cDNA is as strong as the cytomegalovirus immediate-early (hCMV) promoter, which is commonly used for the expression of transgenes in mammalian cells [26]. On the other hand, the dengue virus 5′ UTR cDNA only exhibits cryptic promoter activity in bacterial cells [27]. In this study, we observed that the promoter activity of arterivirus 5′ UTRs is relatively weak, as the luciferase signals from cells transfected with a reporter plasmid carrying arterivirus 5′ UTR cDNA sequences were only in the range of 8- to 10-fold higher than for cells transfected with the empty plasmid. It is not clear if the promoter activity of the 5′ UTR cDNA sequences of these RNA viruses play any biological role in their replication cycle due to the fact that, under natural conditions, these 5′ UTRs only exist in their RNA form. Furthermore, it has been observed that the leaky promoter activity of the dengue virus 5′ UTR cDNA poses a significant challenge for the development of an infectious cDNA clone, due to clone’s instability [27].

## 5. Conclusions

We observed in this study that arterivirus 5′ UTR and PRRSV TRS-B, in their cDNA form, exhibit cryptic eukaryotic promoter activity.

## Figures and Tables

**Table 1:** Primer pairs used in the construction of pNCV1 cDNA clone.

Primer	Sequence (5′→3′)	Application
*Not*I-1F	GCTGCGGCCGCATGACGTATAGGTGTTGGCTC	PCR amplification for pNCV1 F1
4788R	GTCAACCACGATCCTGC	
4004F	CTTAGGCTTGGCATCGTTTC	PCR amplification for pNCV1 F2
8684R	CAGTATTGCGGGAAGAAGA	
8096F	GTGAAGATGCTGCATTGAGAG	PCR amplification for pNCV1 F3
12752R	CACAGCTATTAGCCATTGC	
11340F	CGACGTCAAAGGCACTAC	PCR amplification for pNCV1 F4
A50R	CCGGTTAATTAACGTTTTTTTTTTTTTTTTTTTTTTTTTTTTTTTTTTTTTTTTTTTTTTTTTTAATTTCGGCCGCATG	

**Table 2:** Primer pairs used in the construction of nsp7 and nsp9 mutants.

Primer	Sequence (5′→3′)	Application
NCV18601F	ATCACCGAGGCTGGAGAACTTGTCGGTG	NCV1EAVnsp7 F1
EAVnsp7R	GCCAATGTTGCAGTGAGACTCTCATGATTCATGCCGCAAG	
EAVnsp7F	TGGGCAAGGGGAGCTATGAAGCTGCAAGGCTTTCCATGGAGC	NCV1EAVnsp7 F2
NCV110875R	AATCAAGGTAATCAAGGACAGATGC	
NCV18601F	ATCACCGAGGCTGGAGAACTTGTCGGTG	NCV1SD0108nsp7 F1
SD0108nsp7R	TAGAGCAGCCGTCAGGGACTCATGATTCATGCCGCAAGAC	
SD0108nsp7F	AGCCTGACAACTGCCTTGAAGCTGCAAGGCTTTCCATGGAGC	NCV1SD0108nsp7 F2
NCV110875R	AATCAAGGTAATCAAGGACAGATGC	
NCV18601F	ATCACCGAGGCTGGAGAACTTGTCGGTG	Δnsp7 F1
Δnsp7R	TCCATGGAAAGCCTTGCAGCCTCATGATTCATGCCGCAAGAC	
Δnsp7F	GTCTTGCGGCATGAATCATGAGGCTGCAAGGCTTTCCATGGAGC	Δnsp7 F2
NCV110875R	AATCAAGGTAATCAAGGACAGATGC	
Δnsp9R	AGAACCCTGTCACGGTTTGGGTTCCCAGTGTCACTAGGGGTC	Δnsp9 F1
NCV19176F	TTAGCACCTATGCATTCCTGCCTCG	
Δnsp9F	GACCCCTAGTGACACTGGGAACCCAAACCGTGACAGGGTTCTCG	Δnsp9 F2
12752R	CACAGCTATTAGCCATTGC	

**Table 3:** Primer pairs/sequences used in the construction of pUC19-TRS-Nluc plasmid.

Primer	Sequence (5′→3′)	Application
pUC19F	ATGGCATGCGCCTGGGGTGCCTAATGAGTGAGC	For the construction of pUC19-Nluc plasmid
pUC19R	TACGAATTCTTAAGCCAGCCCCGACACCCGCCAAC	
NlucF	ATGGAATTCATGAACTCCTTCTCCACAAGC	
NlucR	TACGCATGCTTACGCCAGAATGCGTTCGCACAG	
TRS2F	TAAGAATTC**CCTCCGGGTCACATCGTTGAACCAACTTTGGGCCTGG***ACTGAAATGAACTCCTTCTCCACAAGC*	PCR amplification for TRS2-Nluc
**TRS3**	TAAGAATTC**GCTTGACAGGGTCAAACGTAACCATAGTGTACAATAGTTCCCTAGACCGGGTGTTTGCTGTTTTCCCGACCACCGGTTCCCGGCCAAAGCTTCATGATTTCCAGCA***ATGAACTCCTTCTCCACAAGC*	Gibson assembly ligation of TRS3-Nluc
TRS4F	TAAGAATTC**TGACGGCGGCAACTGGTTTCACCTAGA***ATGAACTCCTTCTCCACAAGC*	PCR amplification for TRS4-Nluc
TRS5F	TAAGAATTC**TGAGGTGGGCAACAGTTTTAGCCTGTCTTTTTGCCATCCTATTGGCGATTTGAATGTTCGGGT***ATGAACTCCTTCTCCACAAGC*	PCR amplification for TRS5-Nluc
TRS6F	TAAGAATTC**GTTCCGCAGCAACTCCTGTAACCAAAGTTTCAGCGGAACA***ATGAACTCCTTCTCCACAAGC*	PCR amplification for TRS6-Nluc
TRS7F	TAAGAATTC**TGTTAAACGAGGAGTGGTAAACCTTGTCAAAT***ATGAACTCCTTCTCCAC*	PCR amplification for TRS7-Nluc

Restriction enzyme sites incorporated into primers for cloning purposes are underlined. TRS sequences in the primers are in bold and overlapping 20 nt sequences of *nluc* gene are italicized. F, forward; R, reverse.

**Table 4:** Primer pairs used in the construction of PRRSV 5′ UTR serial 30 nt deletion mutants.

Primer	Sequence (5′→3′)
Δ1-30F	GAATTCTTAAGCCAGCCCCGACA
Δ1-30R	ACATTTGTATTGTCAGGAGCTG
Δ31-60F	CATGGCATAGAGCCAACACC
Δ31-60R	GGCACAGCCCAAAACTTGCCG
Δ61-90F	AGTAGTCACAGCTCCTGACA
Δ61-90R	GCCCTTCTGTGACGGCCTC
Δ91-120F	GTTTCCGCGCGGCAAGTTTTGG
Δ91-120R	TTTAGGGGTTTGTCCCTAACACC
Δ121-160F	CTCCCTTGAAGGAGGCCGTC
Δ121-160R	CGGAGTTGCACTGCTTTAC
Δ161-171F	GAAGCAAGGTGTTAGGGACA
Δ161-171R	CCCTTTAACCATGAACTCCT
Δ5′ UTR(LS)-F	ATGCTCGAGATGAACTCCTTCTCCACAAGC
Δ5′ UTR(LS)-R	ATGCTCGAGAGGGGTGGAGAGACCGTAAAGC
